# Comorbidity, lifestyle factors, and sexual satisfaction among Chinese cancer survivors

**DOI:** 10.1002/cam4.4118

**Published:** 2021-07-13

**Authors:** Jingya Wang, Jie Zhao, Chenggang Zhang, Yuxin Zhang, Nan Jiang, Xiaomin Wei, Jiwei Wang, Jinming Yu

**Affiliations:** ^1^ Key Lab of Health Technology Assessment of National Health Commission and Key Lab of Public Health Safety of Ministry of Education School of Public Health Fudan University Shanghai China; ^2^ Shanghai Xuhui Centre for Disease Control and Prevention Shanghai China; ^3^ Shanghai Health Promotion Center Shanghai China

**Keywords:** cancer survivors, comorbidity, lifestyle factors, sexual satisfaction

## Abstract

**Objectives:**

This study aims to explore the prevalence of sexual satisfaction among Chinese cancer survivors, and explore the association of sexual satisfaction with comorbidity and lifestyle factors.

**Methods:**

A cross‐sectional study was performed among 3996 Chinese cancer survivors recruited at Shanghai Cancer Rehabilitation Club from March to April 2017. Data were collected through self‐reported questionnaires. The questionnaire includes information about demographic, cancer characteristics, comorbidities, lifestyle factors, and sexual satisfaction. Sexual satisfaction was measured by a single‐item scale. The distribution of sexual satisfaction among different demographic and cancer characteristics was compared using the chi‐squared test. Logistic regression models were conducted to assess the effects of lifestyle factors, comorbidities on sexual satisfaction after adjustment for demographic and cancer characteristics.

**Results:**

More than 40% of male and female cancer survivors reported no sexual satisfaction. Sexual satisfaction of cancer survivors is significantly associated with both the number and the type of comorbidities. Heart disease, musculoskeletal system disease, diabetes, and hyperlipidemia are the comorbidities significantly associated with sexual satisfaction of cancer survivors. Lifestyle factors other than smoking, including exercise or fitness, drinking alcohol, and eating fruits and vegetables are significantly correlated with sexual satisfaction. Besides, all of the above associations show gender differences. In addition, demographic characteristics include sex, age, marital status, living status, and average monthly income are also significantly associated with sexual satisfaction of cancer survivors.

**Conclusion:**

Comorbidity and lifestyle factors are associated with sexual satisfaction of cancer survivors, and the associations show gender differences. Improving the lifestyles of cancer survivors, and controlling and reducing their comorbidities are important for improving their sexual satisfaction.

## Abstract 2

This study aims to investigate the prevalence of sexual satisfaction among Chinese cancer survivors, and explore the association of sexual satisfaction with comorbidity and lifestyle factors. Comorbidity and lifestyle factors are associated with sexual satisfaction of cancer survivors, and the associations show gender differences. Improving the lifestyles of cancer survivors, and controlling and reducing their comorbidities are important for improving their sexual satisfaction.

## 1 INTRODUCTION

In 2012, 14.1 million new cancer cases, 8.2 million cancer deaths, and 32.6 million people living with cancer (within 5 years of diagnosis) around the world. The estimated number of new cancer cases will increase to 24 million by 2035.^1^ About 20% of global cancer cases have occurred in China.^2^ Besides, cancer patients diagnosed between 2012 and 2015 had an age standardized 5‐year relative survival rate of 40.5% in China.^3^ Although the primary clinical goal of cancer patients is to control disease, improving the health‐related quality of life (HRQoL) of cancer survivors after treatment is also important, especially when the rate of survival has increased.^3^, ^4^, ^5^, ^6^ As the primary outcome measure in the assessment of sexual health, sexual satisfaction is a key component of the quality of life. Besides, sexual dissatisfaction indicated poor sexual health. The above provides conditions for further investigation of sexual health.^7^

Lawrance and Byers defined sexual satisfaction as “an affective response arising from one's subjective evaluation of the positive and negative dimensions associated with one's sexual relationship”.^8^ A previous study indicated that, factors including the loss of a long‐term sexual partner, deterioration in a continuing relationship, and changes in hormonal status are associated with reduced sexual satisfaction.^9^, ^10^ Besides, declining physical and mental health, and medications for ill‐health are the key factors on reduced sexual satisfaction. Many randomized controlled trials have shown that, compared with control group, male and female with cancer diagnoses were less satisfied with their sex lives. ^11^, ^12^A study in older age group compared the sexual satisfaction among colon and rectal cancer survivors with cancer‐free control found that cancer survivors reported lower sexual satisfaction than controls.^13^ All in all, cancer survivors commonly emphasized that sexual satisfaction is still one of the “unmet needs” to them.^13^

Comorbidity, with the definition of “any distinct clinical entity that has existed or that may occur during the clinical course of a patient with an index disease under study.” It must be distinguished from complications caused by cancer or cancer treatment.^14^ Comorbidity has direct impact on patient care, initial treatment chosen, and evaluation of treatment effectiveness.^16^ Besides, comorbidity is closely related to sexual function. For example, the relationship of erectile dysfunction (ED) with the comorbidities has been broadly evaluated.^13^, ^16^, ^17^ Female sexual dysfunction (FSD) is also found to be associated with comorbidities.^18^ A study in Britain have shown comorbidities are significantly associated with sexual activity and sexual satisfaction of male and female aged 16–74 years, after adjusting for age and relationship status.^19^ A Spain study among male (n = 719, aged ≧50 years) visiting the GP for any medical reasons also shows after age adjustment, participants with comorbidities significantly increased the likelihood of sexual dissatisfaction.

Survivors with cancer often have one or more comorbidities. In many cancers, comorbidities are more important to patient care than cancer itself.^20^ For cancer survivors, comorbidities have been proved to be associated with poorer quality of life.^21^ Some studies indicated that, among cancer survivors, compared with sociodemographic and cancer factors, comorbidities explain more variance in physical and emotional function, pain, and fatigue.^22^ However, the influence of comorbidity on cancer survivors’ sexual health is an area less studied specifically. Given the existing evidences of the relationships between comorbidities and sexual function and sexual satisfaction among cancer‐free population, these evidences deserves a closer examination among cancer survivors.^7^

In addition to the comorbidity, many studies have confirmed that lifestyle factors are closely related to cancer survivors’ quality of life. For example, cessation of smoking and drinking, increased in physical activity, and optimal fruit/vegetable intake are crucial for improving the quality of life of cancer survivors.^23^, ^24^ Although the relationships of lifestyle factors with sexual satisfaction are also less studied among the population of cancer survivors, the relationships of lifestyle factors with sexual function have been extensively studied among cancer‐free population. For example, many studies have shown that physical activity was associated with sexual satisfaction, impotence, ED, and FSD in cancer‐free population.^25^ Alcohol intake is also associated with sexual satisfaction of healthy men.^26^ Smoking was found to be associated with sexual desire among breast cancer survivors,^27^ and was also associated with ED.^28^ It is found that participants without ED were more likely to adopt a Mediterranean diet pattern, which includes fruits, vegetables, nuts, whole grains, and fish, but limited intake in red and processed meat and refined grains.^29^

In the context of Chinese culture, Chinese cancer survivors generally regard sex as a private affair and are hesitant to ask for help from professionals for sexual problems. Consequently, less is known about their sexual life status and the factors which are associated with their sexual satisfaction.^30^, ^31^, ^32^, ^33^ Therefore, this study aims to explore the prevalence of sexual satisfaction among Chinese cancer survivors, and explore the association of sexual satisfaction with comorbidities and lifestyle factors.

## 2 METHODS

### 2.1 Participants and procedures

This study was a cross‐sectional population‐based study. Participants were recruited from 17 multi‐community cancer rehabilitation centers, all of which were affiliated to the Shanghai Cancer Rehabilitation Club (SCRC).^34^ There is a total of about 450,000 cancer survivors in Shanghai, and about 15,000 registered cancer survivors in SCRC. The inclusion criteria of this study included that: 1) has a primary cancer; 2) be able to participate in club activities independently; 3) has no cognitive impairment; and 4) has a basic ability to read and understand. Participants who meet the inclusion criteria will be included in the study. A total of 13,500 registered cancer survivors were judged to meet the inclusion criteria. In this study, the sample size calculation was based on the estimation of 57.4% of cancer survivors who reported being dissatisfied with their sex life.^35^ Under the absolute error of 3%, considering the low response rate of 52.94% of sexual satisfaction item in FACT‐G in previous study,^36^ it was estimated that at least 1,972 cancer survivors should be included into the study.

Between March and April 2017, the survey invitations were sent through WeChat or/and phone calls to those 13,500 registered cancer survivors. Among all these cancer survivors, 1647 were unable to reach because of deaths, migration, or refusing to response; 2728 failed to participate the survey either because they had no time, or because their health status or literacy ability was too poor. Finally, 9,125(67.6%)cancer survivors participated in this survey and completed the questionnaire. Answers to questionnaires were collected in the community activity center affiliated SCRC, through face‐to‐face interviews with the help of well‐trained investigators or through self‐administered questionnaires for literate participants. Each participant was given a tube of toothpaste as a gift for their participation. The questionnaire in the survey was an integrated questionnaire that was used to investigated participants’ demographic information and health‐related conditions and behaviors. Of the returned questionnaires, a total of 3,996 participants completed the item about sexual satisfaction, with a response rate of 43.8%. A total of 5,129 participants failed to complete the item about sexual satisfaction either because they considered sex a private matter, or they ignored this single‐item in the process of filling out the long questionnaires by themselves. Prior to the main study, we conducted a pilot study with 10 cancer survivors from SCRC in order to check the readability and understandability of the questionnaire. According to the results of the pilot study, we modified the semantics and tone of some items in the questionnaire.

The study was approved by the Medical Research Ethics Committee of the School of Public Health, University (The international registry NO. IRB00002408 & FWA00002399). Each of the participants in this study signed an informed consent.

### 2.2 Measurement

A self‐reported questionnaire was developed to collect data of the demographic and cancer characteristics, comorbidities, lifestyle factors, and sexual satisfaction. Demographic characteristics included age, sex, education attainment, marital status, average monthly income, and living status. Cancer characteristics included the type and the course of primary cancer. Comorbidities measured in this study included hypertension, hyperlipidemia, hyperuricemia, diabetes, heart disease, stroke, respiratory disease (such as COPD and asthma), hepatobiliary gastrointestinal system diseases (such as gallstones and fatty liver), and musculoskeletal system diseases (such as osteoarthritis and rheumatoid arthritis). The participants answered whether they lived with any of the above comorbidities. Lifestyle factors measured in this study included smoking, drinking, engaging in physical activity, eating vegetables, and eating fruits. All lifestyle factors were measured by dichotomous questions. Smoking was measured by the question, “Have you smoked more than 100 cigarettes in your life?”; Drinking was measured by the question, “In the past month, have you ever drunk alcohol (such as white wine, red wine, yellow rice wine, beer, wine, etc.)?”; Engaging in physical activity was measured by the question, “In the past month, have you ever participated in any physical exercise or fitness activity (such as running, walking, doing exercises, etc.)?”; Eating vegetables was measured by the question, “Do you eat more than 250 g vegetables a day?”; Eating fruits was measured by the question, “Do you eat fruit every day?”.

Sexual satisfaction was measured by a single‐item scale “I am satisfied with my sexual life” which derived from the authorized Chinese version of Function assessment of cancer therapy‐General (FACT‐G).^37^ The answers of the single item are 0 (“not at all”), 1 (“a little bit”), 2 (“somewhat”), 3 (“quite a bit”) or 4 (“very much”). The evaluation of scores for the single‐item scale had no existing gold standard approach to identify low sexual satisfaction in population samples. So in this study, we treated the “0” score as no sexual satisfaction and deemed that those having a score of “1” or more were satisfied with their sex life.

### 2.3 Statistical analysis

Numbers and percentages were calculated for categorical variables. Chi‐squared test was used to compare the distribution of sexual satisfaction among different characteristics. Logistic regression model was used to calculate adjusted odds ratios (AORs) for associations of sexual satisfaction with comorbidity and lifestyle factors, after adjusting for demographic and cancer characteristics.

## 3 RESULTS

Table 1 reports the distribution of demographic and cancer characteristics, comorbidity, and lifestyle factors for the cancer survivors. A total of 3996 cancer survivors, including 1299 males (32.5%) and 2697 females (67.5%) were included in this study. The average age of the males was 63.91 and that of the females was 58.58. Most of the male participants (92.7%) and female participants (91.8%) were married. More than 80% of male and female participants lived with their spouses. Only a minority of male (8.2%) and female (3.5%) participants had a college degree or higher. More than half of the male and female participants had an average monthly income of 1,000–3,000 RMB (≈140‐420USD). Lung cancer (15.2%), colorectal cancer (24.2%), and gastric cancer (19.8%) were the major cancer types among male cancer survivors. Breast cancer (54.8%) was the leading cancer type among female cancer survivors. More than 78.5% of cancer survivors reported they had one or more comorbidities.

**TABLE 1:** Demographic, comorbidity, and lifestyle factors characteristics, by sex

Variables	Male (n = 1299,32.5%)		Female (n = 2697,67.5%)	
	Number	Percentage (%)	Number	Percentage (%)
**Demographic characteristics**				
Age (years)				
<45	18	1.4	105	3.9
45–54	131	10.1	668	24.8
55–64	567	43.6	1339	49.6
65–74	435	33.5	492	18.3
≥75	148	11.3	93	3.4
Marital status				
Married	1204	92.7	2476	91.8
Others (Single, divorced, and widowed)	95	7.3	221	8.2
Living status				
Living with a spouse	670	50.8	1131	41.9
Living with family	87	5.9	242	9.0
Living with spouse and family	480	36.3	1185	43.9
Living alone	62	4.0	139	5.2
Education				
Junior high school and below	533	40.9	1331	49.4
Senior high school	657	50.4	1269	47.1
College and above	109	8.2	97	3.5
Average monthly income				
<1000	125	9.3	349	12.9
1000–3000	790	60.5	2018	74.8
3000–5000	334	25.4	282	10.5
>5000	50	3.5	48	1.8
**Cancer characteristics**				
Course of cancer				
0–3	286	21.2	557	20.7
4–6	377	28.3	778	28.8
7–9	302	22.5	702	26.0
≥10	334	25.0	660	24.5
Type of cancer				
Lung cancer	198	15.2	126	4.7
Breast cancer	14	1.1	1477	54.8
Colorectal cancer	314	24.2	288	10.7
Gastric cancer	257	19.8	173	6.4
Other cancer types	516	39.7	633	23.5
**Comorbidity characteristics**				
**Number of comorbidities**				
0	279	21.5	612	22.7
1	283	21.8	588	21.8
2	269	20.7	568	21.1
3	196	15.1	373	13.8
4	126	9.7	254	9.4
≥5	146	11.2	302	11.2
**Types of comorbidities**				
Hypertension				
No	811	62.4	1842	68.3
Yes	488	37.6	855	31.7
Hyperlipidemia				
No	920	70.8	1870	69.3
Yes	379	29.2	827	30.7
Hyperuricemia				
No	1161	89.4	2512	93.1
Yes	138	10.6	185	6.9
Diabetes				
No	1066	82.1	2306	85.5
Yes	233	17.9	391	14.5
Heart disease				
No	1016	78.2	2117	78.5
Yes	283	21.8	580	21.5
Stroke				
No	1204	92.7	2525	93.6
Yes	95	7.3	172	6.4
Respiratory diseases				
No	1067	82.1	2376	88.1
Yes	232	17.9	321	11.9
Hepatobiliary gastrointestinal system diseases				
No	686	52.8	1321	49.0
Yes	613	47.2	1376	51.0
Musculoskeletal system diseases				
No	995	76.6	1819	67.4
Yes	304	23.4	878	32.6
**Lifestyle factors**				
Smoking exceeds 100 in whole life				
No	587	45.2	2639	97.8
Yes	712	54.8	58	2.2
Engaging in exercise and fitness in the past month				
No	373	28.7	814	30.2
Yes	926	71.3	1883	69.8
Drink alcohol in the past month				
No	883	68.0	2503	92.8
Yes	416	32.0	194	7.2
Eating vegetables a day				
≤250 g	792	61.0	1684	62.4
>250 g	507	39.0	1013	37.6
Eating fruits				
Not every day	698	53.7	1064	39.5
Every day	601	46.3	1633	60.5

Note

Descriptive statistics were carried out using the number and percentage for the categorical variables.

Table 2 shows the results of the associations between demographic and cancer characteristics and sexual satisfaction. More than 40% of males and females reported no sexual satisfaction. Age, marital status, and living status are significantly associated with sexual satisfaction for both males and females, while average monthly income is significantly associated with sexual satisfaction only for females.

**TABLE 2:** Sexual satisfaction in relation to demographic and cancer characteristics (chi‐squared test), by sex

Variables	Male (n = 1299)			Female (n = 2697)		
	Reporting no sexual satisfaction/ N(%)	Reporting sexual satisfaction/ N(%)	*p* value	Reporting no sexual satisfaction / N(%)	Reporting sexual satisfaction/N(%)	*p* value
All	555(42.7)	744(57.3)		1135(42.1)	1562(57.9)	
Age (years)			**<0.0001**			**<0.0001**
<45	7(38.9)	11(61.1)		31(30.1)	72(69.9)	
45–54	34(26.0)	97(74.0)		212(31.8)	454(68.2)	
55–64	213(37.6)	354(62.4)		581(43.5)	756(56.5)	
65–74	213(49.0)	222(51.0)		249(50.8)	241(49.2)	
≥75	88(59.9)	59(40.1)		60(65.9)	31(34.1)	
Marital status			**<0.0001**			**<0.0001**
Married	496(41.2)	708(58.8)		987(39.9)	1489(60.1)	
Others (Single, divorced, and widowed)	59(62.1)	36(37.9)		148(67.0)	73(33.0)	
Living status			**<0.0001**			**<0.0001**
Living with a spouse	295(44.7)	365(55.3)		468(42.4)	636(57.6)	
Living with family	48(62.3)	29(37.7)		123(56.7)	94(43.3)	
Living with spouse and family	162(34.5)	308(65.5)		418(36.0)	742(64.0)	
Living alone	31(59.6)	21(40.4)		83(72.8)	31(27.2)	
Education			0.782			0.726
Junior high school and below	221(41.6)	310(58.4)		559(42.2)	765(57.8)	
Senior high school	285(43.5)	370(56.5)		534(42.2)	730(57.8)	
College and above	47(43.9)	60(56.1)		35(38.0)	57(62.0)	
Average monthly income			0.912			**<0.0001**
<1000	51(42.1)	70(57.9)		102(30.9)	228(69.1)	
1000–3000	339(43.1)	447(56.9)		881(44.1)	1117(55.9)	
3000–5000	140(42.4)	190(57.6)		110(41.8)	153(58.2)	
>5000	17(37.8)	28(62.2)		11(37.9)	18(62.1)	
Course of cancer			0.715			0.281
0–3	125(45.3)	151(54.7)		242(45.5)	290(54.5)	
4–6	159(43.3)	208(56.7)		300(40.1)	449(59.9)	
7–9	119(40.8)	173(59.2)		285(42.1)	392(57.9)	
≥10	136(41.8)	189(58.2)		265(41.7)	370(58.3)	
Type of cancer			0.816			0.333
Lung cancer	84(42.4)	114(57.6)		54(42.9)	72(57.1)	
Breast cancer	8(57.1)	6(42.9)		597(40.4)	880(59.6)	
Colorectal cancer	138(43.9)	176(56.1)		122(42.4)	166(57.6)	
Gastric cancer	107(41.6)	150(58.4)		75(43.4)	98(56.6)	
Other type of cancer	218(42.2)	298(57.8)		287(45.3)	346(54.7)	

Note

The distribution of sexual satisfaction among different demographic characteristics (including age, education attainment, marital status, living status, and average monthly income) and cancer characteristics (including course of cancer and type of cancer) was compared using the chi‐squared test, by sex.

Table 3 shows the results of the associations between sexual satisfaction and comorbidity and lifestyle factors after adjustment for demographic and cancer characteristics. In both sexes, those reporting more number of comorbidities are significantly to report lower sexual satisfaction. In terms of types of comorbidities, heart disease and musculoskeletal system disease are significantly associated with reduced sexual satisfaction for both sexes. Participants who had diabetes were significantly reported to have reduced sexual satisfaction only for males. Participants who had hyperlipidemia were significantly reported reduced sexual satisfaction only for females. Besides, hypertension, hyperuricemia, stroke, respiratory diseases, and diseases of hepatobiliary gastrointestinal system are not associated with sexual satisfaction.

**TABLE 3:** Sexual satisfaction in relation to comorbidity and lifestyle factors characteristics (logistic regression analysis), by sex

Variables	Male (n = 1299)			Female (n = 2697)		
	Reporting no sexual satisfaction/ N(%)	AOR	*p* value	Reporting no sexual satisfaction/ N(%)	AOR	*p* value
**Comorbidity**						
**Number of comorbidities**		0.925(0.865,0.989)	**0.022**		0.923(0.879,0.968)	**0.001**
**Types of Comorbidities**						
Hypertension			0.384			0.827
No	329(40.6)	1.000		749(40.7)	1.000	
Yes	226(46.3)	0.896(0.699,1.148)		386(45.1)	0.980(0.815,1.177)	
Hyperlipidemia			0.658			**0.006**
No	382(41.5)	1.000		735(39.3)	1.000	
Yes	173(45.6)	0.943(0.726,1.224)		400(48.4)	0.773(0.643,0.928)	
Hyperuricemia			0.275			0.955
No	502(43.2)	1.000		1047(41.7)	1.000	
Yes	53(38.4)	1.238(0.844,1.818)		88(47.6)	0.990(0.709,1.384)	
Diabetes			**0.033**			0.107
No	431(40.4)	1.000		934(40.5)	1.000	
Yes	124(53.2)	0.716(0.527,0.973)		201(51.4)	0.716(0.527,0.973)	
Heart disease			**0.047**			**<0.0001**
No	408(40.2)	1.000		839(39.6)	1.000	
Yes	147(51.9)	0.744(0.556,0.996)		296(51.0)	0.686(0.559,0.842)	
Stroke			0.212			0.243
No	506(42.0)	1.000		1043(41.3)	1.000	
Yes	49(51.6)	0.749(0.476,1.179)		92(53.5)	0.816(0.580,1.148)	
Respiratory diseases			0.143			0.275
No	439(41.1)	1.000		985(41.5)	1.000	
Yes	116(50.0)	0.791(0.578,1.082)		150(46.7)	0.864(0.664,1.124)	
Hepatobiliary gastrointestinal system diseases			0.410			0.216
No	278(40.5)	1.000		539(40.8)	1.000	
Yes	277(45.2)	0.904(0.712,1.148)		596(43.3)	0.900(0.761,1.064)	
Musculoskeletal system diseases			**0.040**			**0.022**
No	399(40.1)	1.000		728(40.0)	1.000	
Yes	156(51.3)	0.743(0.561,0.986)		407(46.4)	0.812(0.680,0.971)	
**Lifestyle factors**						
Smoking exceeds 100 in whole life			0.649			0.394
No	268(45.7)	1.000		1116(42.3)	1.000	
Yes	287(40.3)	1.058(0.829,1.351)		19(32.8)	1.289(0.720,2.308)	
Engaging in exercise and fitness in the past month			**0.020**			**<0.0001**
No	180(48.3)	1.000		400(49.1)	1.000	
Yes	375(40.5)	1.370(1.052,1.785)		735(39.0)	1.394(1.159,1.676)	
Drinking alcohol in the past month			**0.002**			0.508
No	404(45.8)	1.000		1057(42.2)	1.000	
Yes	151(36.3)	1.516(1.172,1.962)		78(40.2)	1.119(0.803,1.559)	
Eating vegetables a day			0.927			**0.025**
≤250 g	339(42.8)	1.000		732(43.5)	1.000	
>250 g	216(42.6)	1.011(0.792,1.291)		403(39.8)	1.220(1.026,1.450)	
Eating fruits			**0.021**			0.056
Not every day	327(46.8)	1.000		432(40.6)	1.000	
Every day	228(37.9)	1.329(1.044,1.692)		703(43.0)	0.843(0.708,1.005)	

Note

Logistic regression models were performed to explore the associations of sexual satisfaction with number and type of comorbidities and lifestyle factors by sex. All models were adjusted for demographic characteristics (including age, education attainment, marital status, living status, and average monthly income) and cancer characteristics (including course of cancer and types of cancer).

Abbreviation: AOR, adjusted odds ratio.

We recorded significant associations between sexual satisfaction and lifestyle factors after adjustment for demographic and cancer characteristics. In males, while individuals reporting engaging in exercise or fitness in the past month and eating fruits every day are more likely to be satisfied with their sexual life; those reporting drinking alcohol in the past month are less likely to be satisfied with their sexual life. Smoking and eating vegetables are not found to be associated with sexual satisfaction among males. In females, individuals reporting engaging in exercise or fitness in the past month and eating vegetables >250 g a day are more likely to be satisfied with their sexual life. Smoking, drinking alcohol, and eating fruits are not found to be associated with sexual satisfaction among females.

## 4 DISCUSSION

The study shows that more than 40% of male and female cancer survivors reported no sexual satisfaction. Sexual satisfaction of cancer survivors is significantly associated with both the number and the type of comorbidities. Heart disease, musculoskeletal system disease, diabetes, and hyperlipidemia are the comorbidities significantly associated with sexual satisfaction of cancer survivors, while hypertension, hyperuricemia, stroke, respiratory diseases, and hepatobiliary gastrointestinal system diseases are not found to be associated with sexual satisfaction. Lifestyle factors other than smoking, including exercise or fitness, drinking alcohol, eating fruits, and vegetables are significantly associated with sexual satisfaction. Besides, all of the above associations show gender differences. In addition, demographic characteristics are also significantly associated with sexual satisfaction among cancer survivors.

The finding of this study revealed that the types of comorbidities are correlated with sexual satisfaction among cancer survivors, which is similar to that of existing studies conducted in cancer‐free population. In a recent study of 719 male in Spain, participants visiting the GP for any medical reasons shows that having comorbidities including hypertension, dyslipidemia, diabetes, or mood disorders significantly reduced sexual satisfaction after adjusting for age.^7^ However, this study indicated that the correlation of hypertension and sexual satisfaction among male and female cancer survivors is not statistically significant after adjustment for demographic and cancer characteristics. The Spanish study also shows that compared with cardiovascular comorbidities (three times greater) or mood disorders (two times greater) alone, the combination of cardiovascular comorbidities and mood disorders influenced a strong effect on sexual dissatisfaction, which is consistent with the finding of this study that the number of comorbidities is associated with sexual satisfaction.^7^ Furthermore, comorbidities are showed to be associated with sexual dysfunction, which largely increased the likelihood of sexual dissatisfaction.^7^ For example, comorbidity has been widely confirmed to be significantly associated with ED.^38^ A study conducted in British females residents aged 16–74 analyzed the third National Survey of Sexual Attitudes and Lifestyles (Natsal‐3), which shows that comorbidities including heart disease, heart attack, stroke, diabetes, hypertension, chronic lung disease, and depression are associated with FSD.^18^ According to a prospective bicentric study,^39^ there is an urgent need for deeper communication between professionals and patients regarding sexual issues to fill the current gap in care of cancer patients.

This study found a positive correlation between physical activity and sexual satisfaction among cancer survivors for both men and women, consistent with existing researches. It is found that physical activity was associated with better sexual satisfaction in cancer‐free population.^40^ Studies have shown that men who maintain physical activity have a lower risk of impotence,^41^ and physical activity is also associated with FSD.^42^ Besides, there is evidence that physical activity is a low‐cost, low‐risk intervention, which can be applied before or in concert with pharmacologic or other treatment and is proved to have a protective effect on erectile dysfunction (ED).^43^ Moreover, physical activity also relieve symptoms for participants who are already suffering from ED.

In this study, alcohol intake is associated with high sexual satisfaction in male cancer survivors. This is in accordance with Emeka's findings that alcohol makes healthy men confident of sexual relationships and could increase sexual satisfaction.^44^ When mentioned intimate partner relationships, alcohol intake with one's partner appears to be an effective way of regulating and relieving negative emotions,^45^ and people drinking with partners reported of increased emotional intimacy the next day.^46^ Besides, alcohol can be used to encourage the reluctant mate or to unleash deviant sexual desires.^47^ The essence of drinking alcohol before engaging in sexual activities is also for pleasurable sex.^44^ In brief, drinking in moderation is a positive factor in sexual satisfaction. However, some studies revealed that the outcomes of alcohol consumption in intimate partner relationships have both negative and positive effects, and alcohol consumption was positively associated with an overall mortality of cancer survivors,^48^ “limited” alcohol consumption is recommended for cancer survivors.

Among breast cancer survivors, smoking was found to be inversely associated with sexual desire, but not significantly associated with sexual satisfaction.^49^ In our study, a link between smoking and sexual satisfaction is also not found. However, the relationship between smoking and sexual dysfunction has been well documented. A study (n = 480) found that smoking is significantly associated with sexual dysfunction in heroin‐dependent patients (HDPs).^50^ Studies also show that smoking could lead to a significant decrease in erection. Moreover, smoking might lead to a decrease in serum testosterone and nitric oxide synthase levels in straight muscle.^28^, ^51^ Other studies also report that smoking is associated with many comorbidities and poor health among adolescent cancer survivors.^52^

Few studies have examined the correlation of diet factors and sexual satisfaction, but some have shown a significant correlation between diet and sexual function. In a case–control study compared male with ED, without diabetes and cardiovascular diseases and male without ED, it was found that the intake of vegetables, fruits, and nuts, and the ratio of monounsaturated lipids to saturated lipids are associated with ED.^29^ Another observational study mentioned that, type 2 diabetic male with the highest adherence to the Mediterranean diet had the lowest prevalence of ED. Moreover, compared with others, they were more sexually active.^53^ A clinical trials revealed that, among participants with obesity or metabolic syndrome, compared with a control diet, Mediterranean diet was more effective in ameliorating ED or restoring absent ED.^29^ In this study, for females, eating vegetables is significantly associated with sexual satisfaction. While for males, eating fruits is significantly associated with sexual satisfaction. This may be explained by sex differences in fruit and vegetable intake. Studies have shown that women rated their liking for vegetables to be higher than for fruits.^54^

This study had several limitations. First, causality between variables cannot be inferred because this study is a cross‐sectional design. Second, participants were sensitive to the question about sexual satisfaction, which may cause selection bias. The response of 43.8% in this study is comparable to the 41.9%–47.6% achieved for studies in Western Australia (N = 4228)^55^and in Brazil (N = 718)^56^ respectively, although higher responses of 54.1%–68.2% have been reported for studies in Japan (N = 170),^57^ Denmark (N = 1045),^58^ and Norway (N = 1271).^58^ Third, the participants were recruited only from SCRC. Future studies with larger or more representative samples are needed to corroborate results of this study. In addition, methodologically we could have chosen a more homogeneous sample, which would have given a concrete idea of the causal factors leading to the lack of sexual satisfaction. This study was conducted on long‐term cancer survivors, and lifestyle factors and chronic comorbidities may be common factors associated with their sexual health. Future studies including more homogeneous cancer survivors are needed to explore more specific sexual health‐related factors for further targeted interventions. Last but not least, our questionnaire relies on self‐reported data, which are subject to measurement bias. Besides, compared with multi‐item scales of sexual satisfaction, a single‐item measure of sexual satisfaction demonstrated the convergent validity, but not test–retest reliability.^59^ A longitudinal study evaluated the sexual quality of life of breast cancer survivors, for the first time to our knowledge, with a specific multidimensional scale dedicated to cancer patients, and pointed out the intervention gaps.^39^ Therefore, compared with multidimensional scales, the single‐item scale measured conclusive sexual satisfaction, which is insufficient in guiding the practice of intervention. It is suggested that future studies on the sexual health of cancer survivors should use a multidimensional scale if response rates can be guaranteed.

## 5 CONCLUSION

Comorbidity and lifestyle factors are associated with sexual satisfaction of cancer survivors, and the associations show gender differences. Improving the lifestyles of cancer survivors, and controlling and reducing their comorbidities are important for improving their sexual satisfaction.

## CONFLICT OF INTEREST

The authors have no funding or conflict of interest to disclose.

## AUTHORS’ CONTRIBUTIONS

Study conception and design: W.J.Y. and Z.J. Data analysis: Z.C.G. and Z.Y.X. Interpretation of results: W.X.M., W.J.W., and Y.J.M Original draft preparation: W.J.Y. and Z.J. Review, editing, and approval of manuscript: all authors.

## ETHICS APPROVAL AND CONSENT TO PARTICIPATE

All procedures performed in studies involving human participants were in accordance with the ethical standards of the institutional and/or national research committee and with the 1964 Helsinki declaration and its later amendments or comparable ethical standards. The study was approved by the Institutional Review Board of the School of Public Health, Fudan University. Ethical approval to conduct this study was granted by the Medical Research Ethics Committee of the School of Public Health, Fudan University (The international registry NO. IRB00002408 & FWA00002399).

## CONSENT TO PARTICIPATE

Verbal informed consent from the survivors for research use of data was obtained before the investigation.

## CONSENT FOR PUBLICATION

After the research, all the survivors gave consent to publication of data and personal information. All authors consented to this publication.

## Data Availability

The datasets analyzed during the current study are not publicly available because consent/approval was not obtained for the sharing of subject data from participants or the Medical Research Ethics Committee of the School of Public Health, Fudan University.
